# Colony geometry and structural complexity of the endangered species *Acropora cervicornis* partly explains the structure of their associated fish assemblage

**DOI:** 10.7717/peerj.1861

**Published:** 2016-04-04

**Authors:** Esteban A. Agudo-Adriani, Jose Cappelletto, Francoise Cavada-Blanco, Aldo Croquer

**Affiliations:** 1 Laboratorio de Ecología Experimental, Departamento de Estudios Ambientales, Universidad Simón Bolivar, Caracas, Venezuela; 2 Grupo de Investigación y Desarrollo en Mecatrónica, Departamento de Electrónica y Circuítos, Universidad Simón Bolivar, Caracas, Venezuela; 3 Laboratorio de Conservación Marino-Costera, Departamento de Estudios Ambientales, Universidad Simón Bolivar, Caracas, Venezuela

**Keywords:** 3-D reconstructions, *Acropora cervicornis*, Fish assemblages, Structural complexity

## Abstract

In the past decade, significant efforts have been made to describe fish-habitat associations. However, most studies have oversimplified actual connections between fish assemblages and their habitats by using univariate correlations. The purpose of this study was to identify the features of habitat forming corals that facilitate and influences assemblages of associated species such as fishes. For this we developed three-dimensional models of colonies of *Acropora cervicornis* to estimate geometry (length and height), structural complexity (i.e., volume, density of branches, etc.) and biological features of the colonies (i.e., live coral tissue, algae). We then correlated these colony characteristics with the associated fish assemblage using multivariate analyses. We found that geometry and complexity were better predictors of the structure of fish community, compared to other variables such as percentage of live coral tissue or algae. Combined, the geometry of each colony explained 40% of the variability of the fish assemblage structure associated with this coral species; 61% of the abundance and 69% of fish richness, respectively. Our study shows that three-dimensional reconstructions of discrete colonies of *Acropora cervicornis* provides a useful description of the colonial structural complexity and may explain a great deal of the variance in the structure of the associated coral reef fish community. This demonstration of the strongly trait-dependent ecosystem role of this threatened species has important implications for restoration and conservation efforts.

## Introduction

Coral reefs are biogenic and highly-diverse ecosystems with high topographic relief and structural complexity (Allemand et al., 2011; Graham & Nash, 2012). The high relief in coral reefs is provided by sessile benthic and modular organisms such as sponges, soft corals and milleporids; and scleractinian corals are the major contributors to the overall structural complexity of these systems (Alvarez-Filip et al., 2009; Coni et al., 2012; Dustan, Doherty & Pardede, 2013; Komyakova, Munday & Jones, 2013). Consequently, these organisms provide habitats for a myriad of associated species including non-sessile invertebrates (Stella, Jones & Pratchett, 2010; Stella et al., 2011; Graham & Nash, 2012) and fish (Risk, 1971; Luckhurst & Luckhurst, 1978; Roberts & Ormond, 1987; Graham & Nash, 2012). Structural complexity has being related to biodiversity in marine and terrestrial habitats (MacArthur & MacArthur, 1961; Luckhurst & Luckhurst, 1978; Gullström et al., 2008). Several studies have indicated that the high biodiversity of coral reefs is strongly related to their structural complexity; for more heterogeneous habitats may increase food availability, provide shelter and, as a result, increase the number of available niches; thereby regulating ecological interactions among coral reef organisms (Birkeland & Neudecker, 1981; Moberg & Folke, 1999; López-Ordaz & Rodríguez-Quintal, 2010; Graham & Nash, 2012).

In the past decade, efforts have focused on determining simple correlations between specific features of the coral habitat and the diversity and abundance of fish communities, particularly at reef-scales (Risk, 1971; Luckhurst & Luckhurst, 1978; Bell & Galzin, 1984; Roberts & Ormond, 1987; Bouchon-Navaro & Bouchon, 1989; Hixon & Beets, 1989; Friedlander & Parrish, 1998; Lirman, 1999; Garpe & Öhman, 2003; Chong-Seng et al., 2012; Komyakova, Munday & Jones, 2013). Results from these studies indicate that the coral and the fish community range from weakly (McManus, Miclat & Palanganas, 1981; Ferreira, Goncalves & Coutinho, 2001; Walker, Jordan & Spieler, 2009; Roberts & Ormond, 1987; McCormick, 1994; Chong-Seng et al., 2012; Komyakova, Munday & Jones, 2013) to highly correlated (Bell & Galzin, 1984; Friedlander & Parrish, 1998; Gratwicke & Speight, 2005). These variable results suggest that the correlations between fish and benthic communities might be naturally variable in space and time (Sale & Douglas, 1984; Ault & Johnson, 1998). Aside from rugosity and topographic relief—two widely used measurements of structural complexity in coral reefs—live coral cover (Bell & Galzin, 1984), coral diversity (Chabanet et al., 1997) and the growth form of coral colonies (Holbrook & Schmitt, 2002) has also shown to influence the abundance, biomass and diversity of the associated fish communities.

Highly variable results reported in the literature may also arise from methodological constraints, as traditional surveys conducted *in situ* might be inadequate to capture the complexity of these benthic habitat structures (Burns et al., 2015a; Burns et al., 2015b). Many of these studies used simple correlations among univariate attributes of fish communities thereby simplifying proxies of benthic structural complexity or coral cover (Luckhurst & Luckhurst, 1978; Reese, 1981; Carpenter et al., 1981; Coker, Graham & Pratchett, 2012) without taking into account the multivariate nature of both the fish and the benthic community.

For example, at reef scales, rugosity has been widely used as a proxy of structural complexity to estimate correlations between the benthic habitat and the abundance (Bell & Galzin, 1984; Bouchon-Navaro & Bouchon, 1989; Coker, Graham & Pratchett, 2012; Friedlander & Parrish, 1998; Reese, 1981), density (Chapman & Kramer, 1999; Ménard et al., 2012), richness and diversity of fish (Carpenter et al., 1981; Bell & Galzin, 1984; Bouchon-Navaro & Bouchon, 1989; Friedlander & Parrish, 1998; Coker, Graham & Pratchett, 2012; Komyakova, Munday & Jones, 2013). However, rugosity focuses on lineal variations in the vertical relief of the reef, by providing a ratio between the length of a chain necessary to reach two separated points in the reef and the actual distance between these two points; while failing to represent the high physical heterogeneity of reef fish habitats. This is because rugosity does not capture important features of the substrate such as the number, area, size and volume of crevices across the sampled benthic area. Previously, measuring the real structural complexity of reef fish habitats was difficult because the variables that best describe this attribute -such as volume of surface area of reef-building organisms-, could not be estimated directly in the field and/or demanded the use of invasive techniques which require the removal of reef organisms (e.g., Lavy et al., 2015). However, recent advances in image analyses have allowed the development of non-invasive methods to determine structural complexity from digital reconstructions, thus improving the accuracy of field estimations (Burns et al., 2015a).

Structure-from-Motion photogrammetry (SfM) (Snavely, Seitz & Szeliski, 2008) is a method that can be used to construct three-dimensional models (3D) used to quantify physical and biological features of coral colonies (Burns et al., 2015a; Burns et al., 2015b; Lavy et al., 2015). This technique generates 3D models of objects or benthic areas from multiple overlapping photographs as input (Westoby et al., 2012). Models created using SfM allow the estimation of structural complexity proxies such as mean surface complexity, slope, and change in curvature; which could be used to explain biological and ecological patterns of variability (Burns et al., 2015a). These models are easy to develop and can be generated without large computational demands. In addition, several geometrical features that contribute to the heterogeneity and complexity of fish habitats can also be measured from these models. Thus, three-dimensional models may be appropriate to assess the relation between fish communities and their habitats using a multivariate approach. This method is particularly useful for fish-habitat associations occurring at small-spatial scales, especially those involving coral colonies with discrete but complex morphologies, body plans and growth forms whose geometric features might be difficult to measure in the field.

In coral reefs, few species provide most of the physical structure that serves as habitat for other invertebrates and fishes. In the Caribbean, *Acropora cervicornis* and *A. palmata* provide habitat for a rich community of fishes and other organisms (Precht et al., 2002). However, by the end of the 1970s, these species populations suffered a substantial decline due primarily to withe band disease (Aronson & Precht, 2001) and since then, their recovery have been hampered by the interaction and positive feedbacks of natural and anthropogenic stresses operating at both, local and global scales (Weil et al., 2002; Precht et al., 2002; Carpenter et al., 2008; National Marine Fisheries Serviceh, 2014). This led to the inclusion of *Acropora cervicornis* in the IUCN Red List of threatened species under the category of critically endangered, and in the US Act of Endangered Species as threatened (Precht et al., 2002; Aronson et al., 2008). Until the downfall of *Acropora cervicornis* populations, this species was the dominant reef-builder at intermediate depths (5–25 m) in shallow fore reefs and lagoonal environments in the Caribbean (Aronson & Precht, 2001; Weil et al., 2002). Thus, understanding the relationship between the physical structure characteristics of this species and the associated fish assemblages from a functional trait perspective might be extremely important to better comprehend its role in the maintenance and delivery of key coral reefs provisioning ecosystem services (Moberg & Folke, 1999; Bouma & Van Beukering, 2015) as well as to provide empirical evidence of its role as a surrogate species (Mumby et al., 2008).

**Figure 1:**
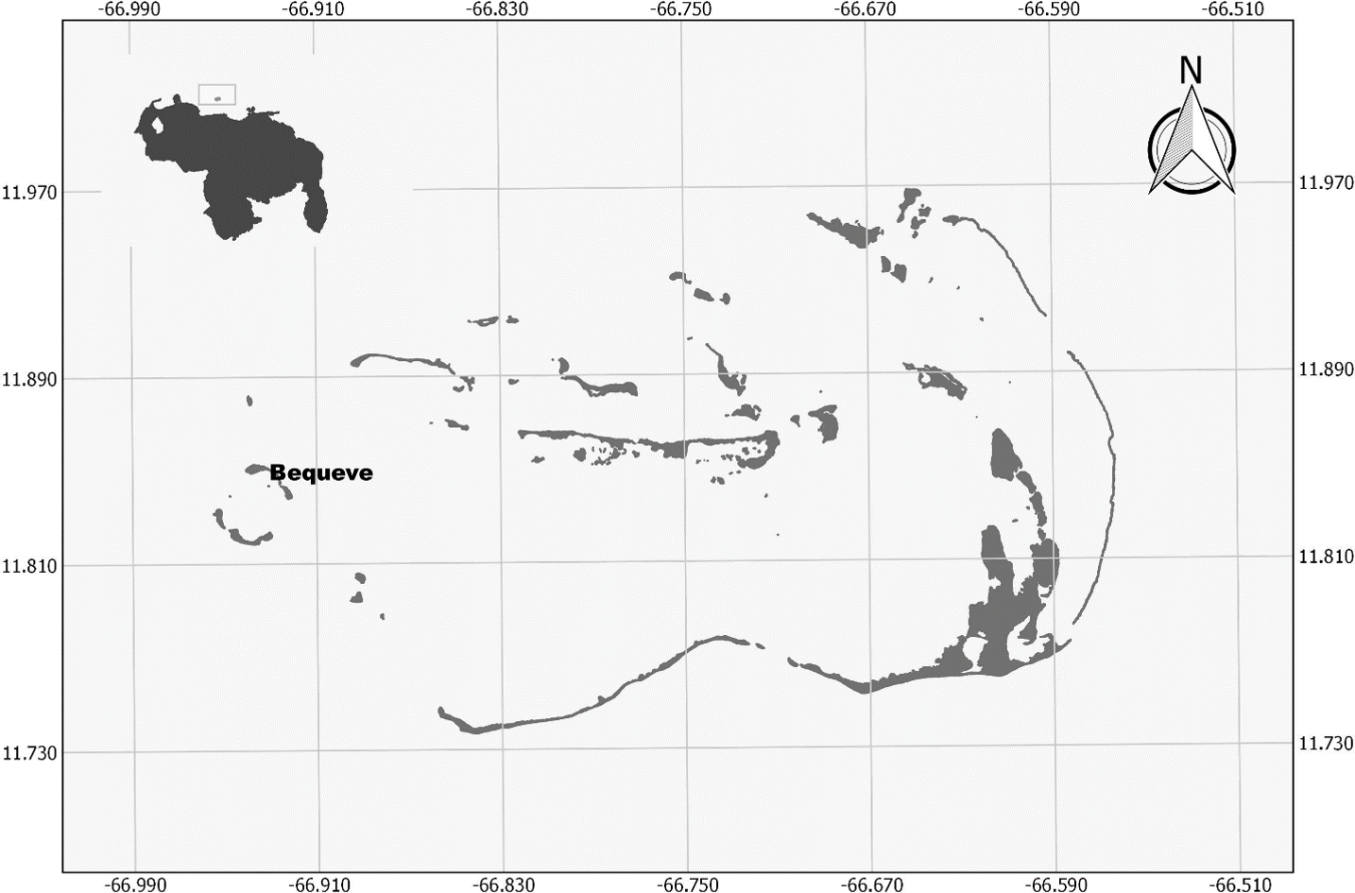
Study site map. Map of the Archipielago de Los Roques National Park, with the study site.

Herein we introduce the use of 3D models of coral colonies to determine their structural complexity (i.e., volume, number of branches, surface, height), geometry (i.e. length, width and height) and their biological features (i.e., live coral tissue, algae and other substrates) from videos, using *Acropora cervicornis* colonies as our study model. We then use these physical and biological features to determine whether the geometry, the structural complexity and/or the biological characteristics of the colonies, explain the variability in the fish assemblage structure at the colony scale.

## Materials and Methods

### Study site

The study was conducted at Archipelago Los Roques National Park (ALRNP) a marine protected area (MPA) located 170 km off the central coast of Venezuela, in the southern Caribbean (Fig. 1). The archipelago covers a total area of 225.2 hectares encompassing more than 50 cays, sand flats, mangrove forests, seagrass beds and coral reefs. This MPA is the most extensive, well-developed coral reef ecosystem in Venezuela, not only in terms of its marine biodiversity and the resources it provides; but also for the high coral cover found on its reefs and the presence of herbivores fish which are becoming overfished in the Caribbean (Posada, Villamizar & Alvarado, 2003; Jackson et al., 2014). Sampling was carried out on the seaward side of Bequeve cay (11°50′36.12″N, 66°55′51.30″O, Fig. 1). This site was chosen because discrete colonies of *Acropora cervicornis* occurred on a bare and extensive sandy bottom covering an area of 265 m^2^, thus reducing the chance that all the observed fishes in this study moves between colonies and other habitats such as mangrove and/or seagrass beds.

### Sampling design

A total of 20 discrete colonies of *Acropora cervicornis* displaying different morphological features (i.e., height, length, thickness and number of branches) were randomly selected at the same depth (1–1.5 m). In order to reduce the possibility of having fishes moving from one colony to another, only colonies that were at least 2 m apart from each other were selected. This criterion was based on direct observations of the fish communities interacting between neighboring colonies. Each of these coral colonies were systematically sampled in three steps: fish counts were carried out first, then length and height of the colony were manually measured *in situ* and finally, a video of the colony was taken by swimming around each colony for at least 2 min. The videos were taken using an Intova Sport HD Edge camera, with a resolution of 1,080 pixels at 30 frames per second. Working with videos instead of pictures is more time efficient as image acquisition is faster and provides a larger number of images for 3D reconstructions. All the surveys were conducted in a single day from 1 to 2 pm in April 2014.

### Characterization of fish assemblages

The fish community was described using visual censuses. For this, each colony was observed for approximately six minutes to identify and count each individual (adult or juvenile) observed at a maximum perimeter of 50 cm around the colony. Juvenile haemulids were identified to genera, as they are often found in large mixed schools, difficulting an accurate identification to species level. Discrimination between adult and juveniles was based on individual coloration patterns.

### Three dimensional reconstructions of *Acropora cervicornis* colonies

**Figure 2:**
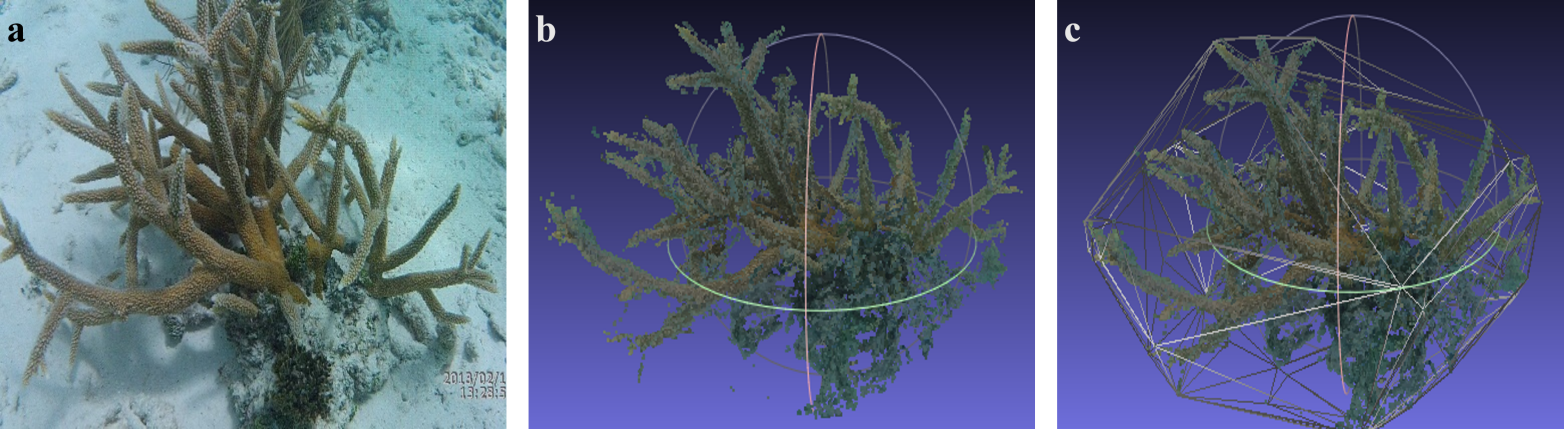
3D model reconstruction. Sequence of pictures depicting the 3D model reconstruction process. (A) Photo extracted from video of *Acropora cervicornis*. (B) 3D reconstruction. (C) 3D reconstruction with convex hull filter.

Three dimensional models for each colony were reconstructed following three steps. First, each video was cut down at a 10 pictures per second rate, thus obtaining 200–400 JPEG pictures per video. For this, the ffmpeg V 2.7 tool was employed. Second, we identified matching points from consecutive sets of 30 pictures using the SiftGPU-V400 (Wu, 2011) software and following the pairwise matching criterion. Third, a sparse three-dimensional point cloud was generated after matching each picture of the set in the previous step. For this, we employed VisualSFM V.0.5.26 (Wu, 2013), where as many points as possible were added, in order to obtain a denser point cloud from which the 3D model was created and then exported in PLY format (see Fig. 2).

**Figure 3:**
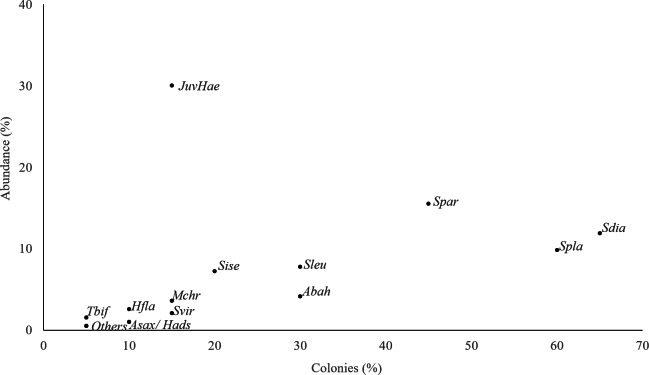
Fish abundance and occurrence. Total abundance of each species and percentage of colonies where each species was observed. JuvHae, Haemulid in juvenile phase; Tbif, *Thalassoma bifasciatum*; Other, *Ocyurus chrysurus*; *Hypoplectrus puella*, *Cantherhines pullus*; Hfla, *Haemulon flavolineatum*; Asax, *Abudefduf saxatilis*; Hads, *Holocentrus adscensionis*; Mchr, *Microspathodon chrysurus*; Svir, *Sparisoma viride*, Sise, *Scarus iseri*; Sleu, *Stegastes leucostictus*; Abah, *Acanthurus bahianus*; Spar, *Stegastes partitus*; Spla, *Stegastes planifrons*; Sdia, *Stegastes diencaeus*.

### Image analyses: measuring variables of structural complexity

The 3D models were analyzed using Meshlab v1.3.3 (Cignoni, Corsini & Ranzuglia, 2008) to estimate in pixels the following geometrical variables: length, height, width, the volume and area of the convex hull, using the software measurement tools. The convex hull filter generates a mesh whose limits are determined by the most external points of the cloud (i.e., vertex). These points are set by the tip of the branches located at the periphery of each colony. Therefore, this function simulates a blanket covering the colony (Fig. 2C). The surface of, and the volume covered by this mesh was then calculated to get a proxy of the three-dimensional structure of each coral colony. Then, for each model we used the length (i.e., maximum distance between opposite sides of a colony) and height (i.e., distance from the base to the taller branch) recorded in the field to scale them in centimeters.

Finally, we defined two indices of structural complexity. The first one was estimated by the ratio between the actual surface of the colony and the surface of the convex hull. To determine the actual surface of each colony, the Ball Pivoting Algorithm was used (Bernardini et al., 1999). This algorithm was chosen because it is based on a mesh built from internal rather than extreme points and so, it provides a closer approximation of the authentic surface of each colony (Bernardini et al., 1999). The second index was defined by the number of branch tips (i.e., vertex) that forms the convex hull, standardized by its area (vertex/m^2^), and thus, was considered a proxy of the number of peripheral branches per unit area.

The percentage of live coral tissue, macroalgae and turf algae were also determined from digital images, for each colony. For this, fifty random points were overlaid onto an image of each of the colonies, identifying the object underneath each point. Percentage of each substrate type was estimated as the number of points over a given category divided by the total number of points (Kohler & Gill, 2006). For each of these colonies, the number of branches (peripheral and internal) per frame (i.e., a 25 cm^2^ square) was also determined. We used 4 frames for colonies smaller than 1 m and 8 frames for larger colonies. We verified that the number of frames being sampled represented 40–50% of each colony’s total area, regardless of their size. All these analyses were performed with the software CPCe V 4.1 (Kohler & Gill, 2006).

### Statistical analysis

A distance-based linear model analysis (DistLM; Legendre & Anderson, 1999; McArdle & Anderson, 2001) was carried out to determine whether the geometrical and biological features of each coral colony explained the variability in the structure, the abundance and the richness of the fish assemblage associated with each colony. This analysis, models the relationships between a multivariate data cloud (i.e., fish assemblage), which is described by a similarity matrix calculated using the Bray Curtis index, and the predictor variables, (i.e., the geometrical and biological features of each colony). The fish matrix was square rooted to weigh down highly abundant fishes, while benthic data was normalized following the procedures outlined by (Clarke & Gorley, 2006). No transformations were performed for the benthic data because no variables were skewed. This model was visualized using a distance-based redundancy analysis (DbRDA), a constrained ordination method in which the axes are directly and linearly related to the predictor variables (McArdle & Anderson, 2001; Clarke & Gorley, 2006). The same approach was used to determine the set of predictor variables that better explained the variability of total abundance and richness of fishes, but using Euclidean distances to construct the similarity matrix.

This study was authorized by the Ministerio del poder popular de Ecosocialismo, Hábitat y Vivienda (Approval number: 0323) and Territorio Insular Miranda (Approval number 006).

## Results

**Figure 4:**
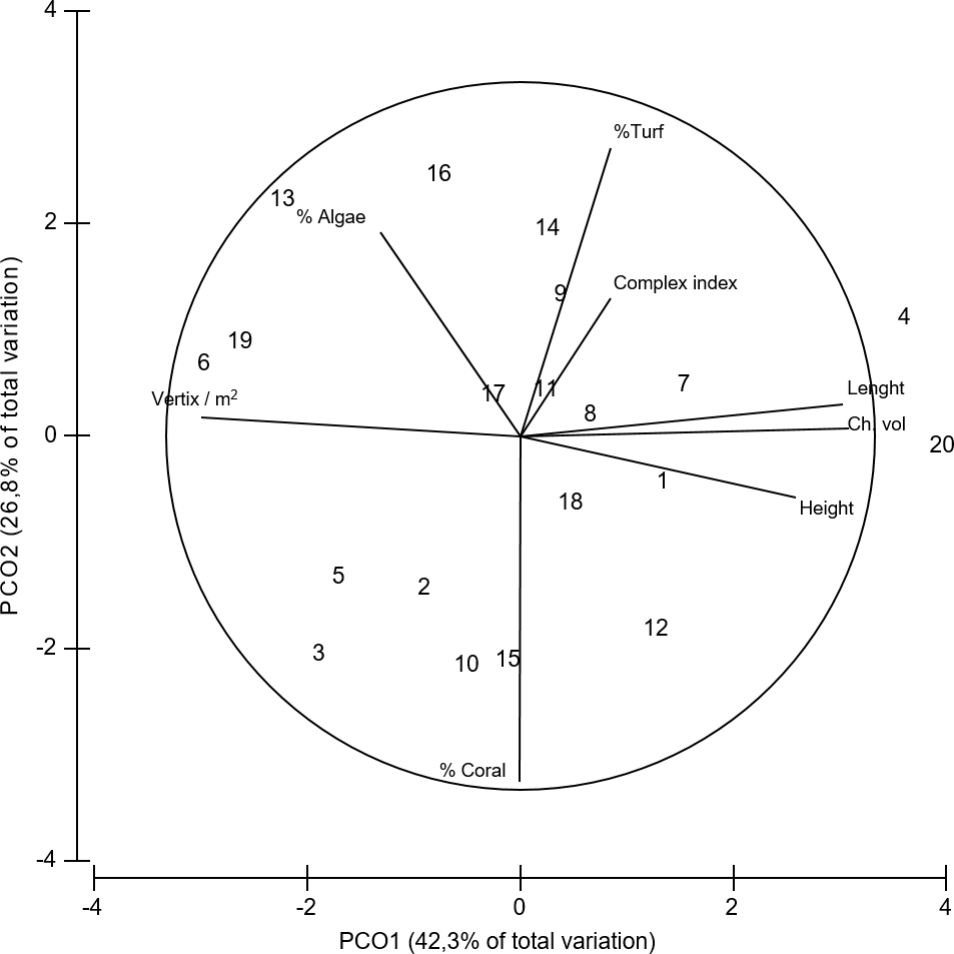
PCoA of coral variables. Principal coordinates analysis (PCoA) of the geometrical and structural complexity and biological variables estimated for *Acropora cervicornis*. Data was previously normalized.

Coral colonies exhibited a high variability in their geometrical and biological characteristics, further indicating that the structure of the fish habitat measured features changes from one colony to the other (Table 1 and Fig. 4.). For example, colony length and height ranged from 50 to 175 cm and from 27 to 80 cm, respectively. Similarly, coral cover was also variable ranging from colonies with no coral cover to colonies with 100% cover of live tissue (Table 1).

**Table 1:** Geometrical and biological variables estimated for the 20 colonies. Range, average and standard deviation of the geometrical and biological variables estimated from the 20 colonies of *Acropora cervicornis* at Baquevé cay, Archipelago Los Roques.

	Lenght (cm)	Height(cm)	Ch. vol (lts)	Complex index	Vertix / m^2^	% Coral	%Turf	% Algae	Av. branches
Max	175.00	80.20	261.70	0.73	509.26	100.00	76.47	62.50	10
Min	50.00	27.00	13.61	0.39	88.78	9.09	0.00	0.00	3
Average	98.30	44.51	87.30	0.56	228.93	58.54	25.08	15.33	7.30
Std. desvt.	36.70	12.05	67.23	0.08	121.53	27.30	21.28	15.70	1.98

**Notes.**

Max maximum value Min minimum value Average arithmetic mean Std Standard Deviation

A total of 193 fish belonging to 11 families and 20 species were found inhabiting *Acropora cervicornis* colonies at our study site. The most abundant group of fish were juveniles of the family Haemulide, accounting for 30% of the total abundance. While this family was abundant, their distribution among the sampled colonies was clumped as they were observed only in 15% of the surveyed colonies (Fig. 3). Species such as *Stegastes diancaeus, Stegastes partitus* and *Stegastes planifroms* showed relative abundances of 18% and 30%, respectively and they were present in 45–65% of the surveyed colonies. Other species such as *Stegastes leucostictus* and *Acanthurus bahianus*, were less conspicuous, and were observed only in 30% of the sampled colonies with relative abundances rarely exceeding 10% (Fig. 3). Thus, the results indicated that there was not a single common group of fishes associated with all the colonies.

**Figure 5:**
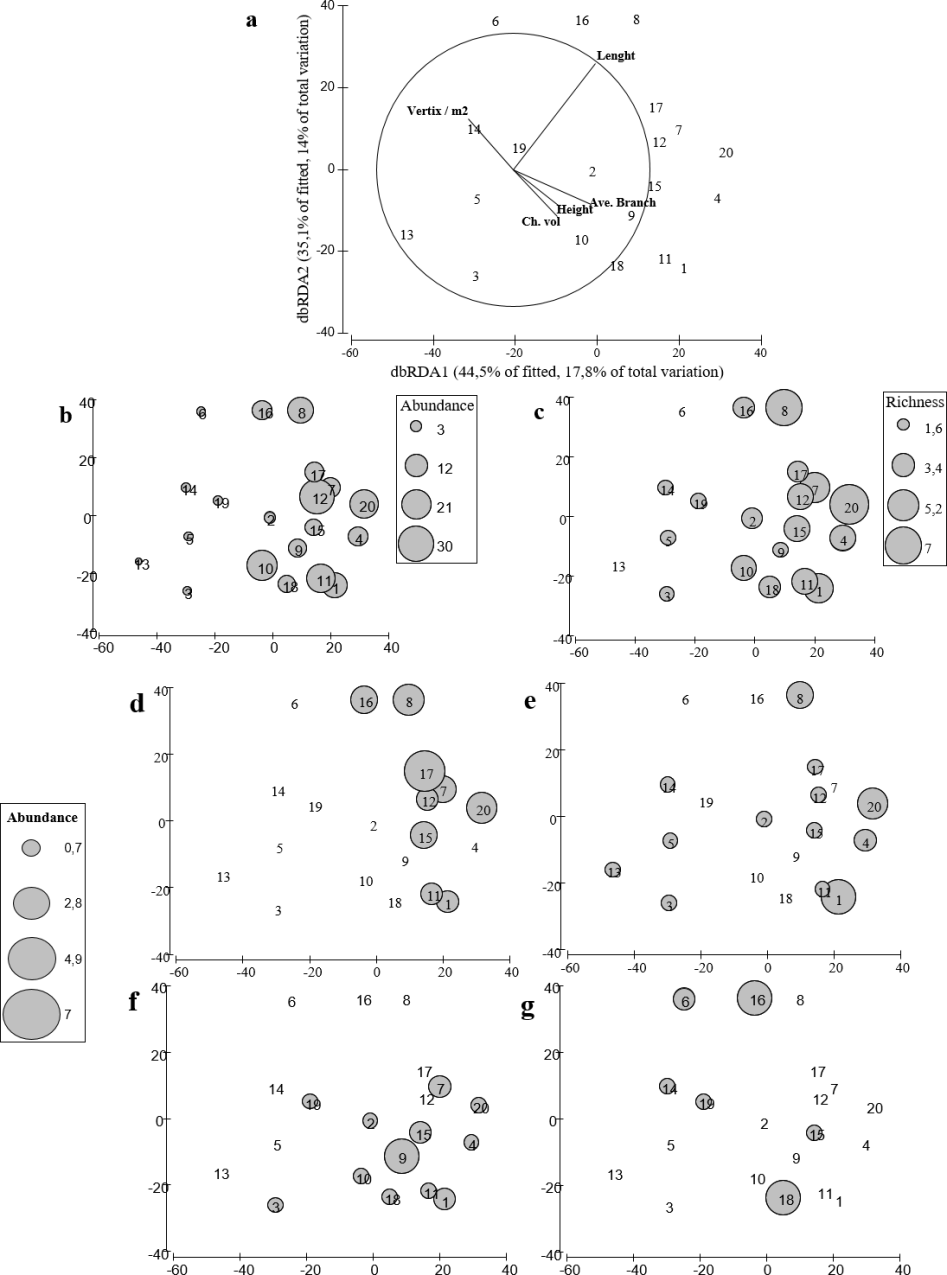
distLM for fish assemblages vs. coral variables. Redundancy analysis based on distance that explains the contribution of each variable to the model that better explains the variability on the fish assemblage. (B, C) Redundancy analysis based on distance with bubble plot representing the abundance and richness of the fish assemblage. (D–G) Bubble plot representing the abundances of the damselfish species found in *A. cervicornis* colonies. (D), *Stegastes partitus*. (E) *Stegastes diancaeus*, (F) *Stegastes planifroms*, (G) *Stegastes leucostictus*.

The distance-based linear model showed that geometrical rather than biological variables explained more variance in the structure of fish assemblage; being the length of the colony the most important explanatory variable (Table 2). The results indicated that nine variables explained up to 53% of the total variability observed in the fish assemblage. Although only length, height, volume, the number of peripheral branches (vertex/m^2^) and the average total number of branches were significantly correlated with the fish assemblage structure (Table 2), these five variables only explained 40% of the observed variability in the fish assemblages (Table 2, Fig. 5A).

**Table 2:** Variables used to construct models of fish assemblages. Explanatory variables used to construct the model that explained most of the variability of the associated fish assemblage.

Variable	Variance explained	*P*-value
Lenght (cm)	18%	0.003^**^
Vertix/m^2^	16%	0.001^**^
Height(cm)	11%	0.019^*^
Ch. vol (lts)	15%	0.022^**^
Av. branches	11%	0.030^**^
% Coral	5%	0.254
% Algae	4%	0.6983
%Turf	4%	0.680
Complex index	5%	0.673
**Correlation of the model whit all the variables**	54%	

**Notes.**

* Statistical significant variables, with alpha <0.05

** alpha < 0.005 minimum.

Our results suggest that colonies with higher complexity supported fish assemblages with higher species richness (Fig. 5B) and higher total abundance (Fig. 5C). Furthermore, colonies with different morphologies also differed in the fish species composition. In fact, geometrical and biological attributes of the coral colonies, explained 61% and 69% of the total variance in fish abundance and richness, respectively. The distribution of the four species of damselfishes -farmer pomacentrid (Ceccarelli, Jones & McCook, 2005)— recorded in our study differed among colonies (Figs. 5D–5G). Among damselfish, *Stegastes planifroms* was the most abundant species and; it was recorded in colonies with varying morphological features. And this species was also observed sharing colonies with *S. partitus* and with *S. diancaeus*. However, the first was observed particularly in larger colonies, while the latter was recorded regardless of the morphological features exhibited by the shared colonies. *Stegastes leucostictus* was the least abundant species and was observed mostly in colonies that were not inhabited by any other of the observed damselfish species (Figs. 5D–5G).

Again, length, convex hull volume, number of peripheral branches and average number of branches were significantly correlated with both total number of species and abundance of fishes. Thus our results suggest that structural complexity measured as a multivariate property of each coral colony may affect the structure of fish assemblage, not only in terms of species richness but also in terms of total abundance. In addition, the complexity of *Acropora cervicornis* seemed to enable the coexistence for only some damselfish species (e.g., *S. planifroms* and *S. partitus*), whereas species such as *S. leucostictus* were only documented in less complex and depauperated colonies. Although coral morphology clearly influenced local fish assemblage’s structure, 30–60% of the total variance in structure, abundances, and richness remained unexplained or not correlated with the geometry and/or the biological features of the coral colonies measured.

## Discussion

Our results indicate that geometrical and biological characteristics of *Acropora cervicornis* may have a striking effect on the associated fish assemblage. Consistently, length, volume, the number of peripheral branches (vertex/m^2^) and the average number of branches, were the most important variables as they explain up to 40% of the variability in the structure, and 61% and 69% of the abundances and richness of the fish assemblage respectively. Results from the present study also suggest that 3D models built from videos in the field allow the quantitative estimation of geometry, size and structural complexity of a highly structurally complex coral species such as *Acropora cervicornis*. This suggests that the method could also be used for coral species with lower structural complexity. Measurements from three-dimensional reconstructions made using Structure from Motion have been proved to be highly accurate when compared with other techniques like wax dipping, modeling using artificial figures of coral, laser scanning, or computerized tomography (Bythell, Pan & Lee, 2001; Courtney et al., 2007; Laforsch et al., 2008; McKinnon, Upcroft & Smith, 2011; Burns et al., 2015a; Lavy et al., 2015). A great advantage that 3D reconstructions from pictures and/or photos have, when compared to other methods, is the low cost and the non-invasive nature of the image acquisition procedure. Nevertheless, it remains to be tested whether this method can be used in larger scales such as reef zones, reef sites or entire reef systems.

Three-dimensional reconstructions for assessing the relationship between fish assemblages and their habitat might be limited by the geomorphological characteristics of the habitat in terms of resolution (Hedley et al., 2016) and the spatial extent of the benthic system in terms of cross-scale patterns (Nash et al., 2014) of both, the habitat structural features and the fish assemblages. The difficulty associated with measuring multiple proxies of structural complexity *in situ*, particularly for reef-seascape studies (Hedley et al., 2016), makes the use of rugosity as a proxy of habitat complexity (Risk, 1971; Luckhurst & Luckhurst, 1978; Friedlander & Parrish, 1998; Ferreira, Goncalves & Coutinho, 2001; Walker, Jordan & Spieler, 2009; Coker, Graham & Pratchett, 2012) still useful to study benthic-fish assemblage relations. However, three-dimensional reconstruction using videos and photographs may solve this problem, especially for studies focused on small spatial scales, for it is non-invasive and retrieves multiple proxies of structural complexity. Nevertheless, the use of 3D reconstructions for establishing the correlation between fish communities and their habitats for larger spatial scales might not be as straightforward as in our study. This is because interactions and behavior between fishes and their habitats may differ from colony to reef scales (Nash et al., 2013).

For example, juvenile and small fishes recorded in this study (e.g., *Stegastes* spp.) seldom move away from their habitat compared to other fishes that frequently move among and within different reef habitats (Robertson & Lassig, 1980). At small spatial scales (e.g., colonies) juvenile phases and/or small fish are highly abundant. These fishes tend to be more territorial with a restricted ability to move across reef sections compared to adult fishes, due to predation avoidance (Werner et al., 1983; Sogard, 1997). Therefore, juveniles and small fishes utilizing discrete colonies of *A. cervicornis* will relate with their habitat differently compared to adult and larger fishes, which move across reef sections and even reef systems (Nash et al., 2013).

Most shallow marine benthic habitats are formed by foundation species, organisms that can modify their environment and generate new habitats (Bruno & Bertness, 2001). This is the case for *A. cervicornis* colonies in Los Roques. This coral species forms monospecific patches of different sizes, providing refuge from predation through the growth of their branches, which contribute to the formation of intricate structures. It is thus expected that structural characteristics of *A. cervicornis* colonies, would be related to fish assemblage attributes. Our results indicate that at Bequevé, colony size seems to be the most important structural feature related to the structure, abundance and species richness of the fish assemblages associated with *Acropora cervicornis* colonies. However, the variables used as proxies of structural complexity (height, convex hull volume, the density of peripheral branches (vertex/m^2^) and the average number of branches) were also highly correlated to the fish assemblages; indicating that species-specific traits of this foundation coral species might be important in determining habitat complexity and therefore abundance, richness and composition of small associated fish assemblages.

Although the importance of species-specific traits of foundation species as modulators of the spatial distribution, composition and persistence of their associated community have been recognized (Angelini et al., 2011), in the case of *Acropora cervicornis*, this might also have big implications for management, *ex situ* conservation and restoration programs. The ecological importance and threatened status of *A. cervicornis* populations has led to the development of numerous restoration programs, many of which focus on coral gardening (Garrison & Greg, 2008; Herlan & Lirman, 2008; Young, Schopmeyer & Lirman, 2012). These programs argue that coral gardening could be a promising alternative to restore lost wild populations (Herlan & Lirman, 2008; Forrester et al., 2014; Lohr et al., 2015) while having a positive impact in the delivery of shallow reefs ecosystem services, through increasing nursery and predation-protection habitats for reef fishes (Mumby et al., 2014). The results of this study provide valuable information that can be taken into account when transplanting corals fragment, as they provide evidence on key structural features related with the function of this coral as a foundation species. The size of the new patches but also the number of branches and the vertical structure of the colony are variables that should be important to consider in both, ex situ and *in situ* restoration programs.

*Acropora cervicornis* structures, full of crevices and holes, serve as refuge for different fish species, some of them being in juvenile phases. A heterogeneous habitat such as this, may facilitate coexistence between competitors within the same colony by increasing the number of available microhabitats and territories in a spatial scale ranging from centimeters to a few meters. This is particularly important for small territorial species such as damselfishes. In our site, Pomacentrids (e.g., *Stegastes partitus*, *S. diencaeus*, *S. planifroms* and *S. leucostictus*) and juvenile grunts (Haemulidae) were the dominant fish groups; accounting for about 80% of the total abundance recorded inside or close to the coral colonies. Damselfishes are known to maintain and defend small territories against congener and/or other species (Sammarco & Williams, 1982; Robertson, 1996; Holbrook, Forrester & Schmitt, 2000; Ceccarelli, Jones & McCook, 2005), whereas juvenile grunts often use corals, seagrasses and mangrove roots has habitats and/or refuges (Negelkerken, 2000). Considering this, it might be possible that colony complexity favors the abundance and richness of associated fish species.

On this sense, our observations also suggest that colonies with too many branches per meter (vertex/m^2^) may form an intricate and tangled mesh that might make the colony unsuitable for some fish species, through a limitation imposed by body size. This might explain why, with the exception of pomacentrid, all observed fishes were in juvenile phases. This finding further illustrates and supports that the morphology of *Acropora cervicornis* plays a central role in determining the abundance and species richness of their associated fish assemblages but also in determining species composition. This have been suggested for many other foundation species in both terrestrial and marine ecosystems (Angelini et al., 2011).

For instance; *Stegastes planifroms* was observed sharing the same colonies as *S. partitus* only when the colonies were larger than 100 cm. *S*. *planifroms* is a large and aggressive species known to compete and displace damselfish like *S. partitus* (Robertson, 1996). Our results suggest that coexistence between these two species is only possible if coral colonies are large and complex. This may be explained as large colonies could release competition pressure by providing enough territory for coexistence. According to Robertson (1996), *S. planifroms* often coexists with damselfish species such as *Stegastes diancaeus* or *Stegastes leucostictus* (Robertson, 1996). Our results suggest that *S. planifroms* and S. *diancaeus* coexist regardless of the size of coral colonies. However, *S. leucostictus* was observed in colonies that were not inhabited by any other damselfish species. *S. leucostictus* has poor site fidelity (McGehee, 1995); therefore it is possible that this species avoided colonies occupied by other fish species.

Even though antagonist interactions like competition and predation, as well as abiotic factors had received the most attention as forces that structure ecological communities, facilitation has being also recognized as important as these other factors, especially during the last decades (Bruno & Bertness, 2001; Bruno, Stachowicz & Bertness, 2003; Bulleri et al., 2016; Bruno & Kennedy, 2000). Facilitation can expand species realized niche trough providing refuge that limits predation (Bulleri et al., 2016). *Acropora cervicornis* could be acting as an indirect facilitator, providing physical structure that allows numerous species of fish to hide from predators and that probably would not inhabit in the same abundance in bare sand patches. Also, larger and more complex colonies have a lot of branches that increase the available surface for territorial damselfish to establish farming territories.

Other processes apart from habitat size and structural complexity may also be important in shaping the structure of these fish communities as 30–45% of the total variance of the fish community remained unexplained. This suggests that factors controlling larval dispersion, settlement and survivorship, which are known to have a central role in structuring coral reef-fish assemblages might be operating at the scales examined in this study (Victor, 1986; Ohman & Rajurisa, 1998).

Surprisingly, we found that live coral cover only explained 4% of the total variance recorded for the fish community associated with *Acropora cervicornis* at our study site. Most of the fish species observed in association with these colonies (i.e., Pomacentrids and Haemulids) are not considered habitat specialist or highly associated to coral cover. Instead, these species are associated to a broad variety of habitats or can change their preferred habitat (Dominici-Arosema & Wolf, 2005; Figueira et al., 2008; Precht et al., 2010). However, live coral cover must be an important component of the habitat for it helps to maintain the structural integrity of the colonies. It is well known that the rapid loss of coral tissue is often accompanied by a rapid colonization of opportunistic organisms such as sponges, calcareous algae, macroalgae and turf algae (Done, 1992; Nyström, Folke & Moberg, 2000). This in turn may reduce spatial complexity by decreasing the number of holes due to obstruction and/or by increasing the bioerosion and abrasion of the carbonate physical structure (Alvarez-Filip et al., 2009).

However, assessing the significance of coral cover lost on the association between fish assemblages and topographic complexity is difficult as these two variables are sometimes highly associated (Komyakova, Munday & Jones, 2013). Most of the monitoring programs focus on estimating coral cover and do not take into account changes in the reef’s structural complexity (CARICOMP, 2001; Hill & Wilkinson, 2004). Even though coral cover is an important variable to estimate degradation on coral reefs (Hughes, 1994; Bellwood et al., 2004), using only this variable could limit the ability to identify changes that could also affect the reefs function and resilience (Graham et al., 2013).

In our study, low variance explained by coral cover contrasts with previous studies conducted across reef patches; which show that coral cover determines fish recruitment (Coker, Graham & Pratchett, 2012), the abundance of fish during early life stages (Wilson et al., 2008) and the abundance of highly specialist reef fishes (Pratchett et al., 2012). Nevertheless, the majority of these studies are not directly comparable with our findings for they were conducted in the Pacific, a bioregion with larger number of coral and fish species and where many fish species have being reported to be highly specialist (Wilson et al., 2008; Burke et al., 2011; Pratchett et al., 2012; Komyakova, Munday & Jones, 2013).

From our results, we conclude that three-dimensional reconstructions represent a valuable tool to measure variables that describe the geometry and the structural complexity of fish habitats, at least at the colony scale. This method allowed the reconstruction of colonies of *Acropora cervicornis*, a coral species with a complex body plan without harming the coral. This suggests that this procedure should also be applicable for benthic organisms with less structural complexity. It is also a low cost technique that requires little time in the field. The methodology employed in this study allowed the quantification of fish-habitat relationships using a multivariate approach. Combined, the structural features of the coral colonies explained 62% of the variance of abundance, 69% of the variance in fish species richness and 40% of the variance in the structure of the fish assemblage associated to this coral species. These results indicate that at least four structural variables (length, convex hull volume, number of peripheral branches and average number of branches are specie-specific traits of *Acropora cervicornis* as a foundation species that might have an effect in the composition and spatial distribution of the associated fish assemblages. We suggest that using multivariate instead univariate approaches to describe fish-habitat correlations, particularly for studies conducted at small spatial scales could be a more comprehensible approach to understand the nature of these relationships.

## Supplemental Information

10.7717/peerj.1861/supp-1 Supplemental Information 1 Raw Data geometry explain fish assemblage Raw data containing the abundance of fishes and the geometrical characteristics of the A. cervicorn is colonies.

## Additional Information and Declarations

### Field Study Permissions

The following information was supplied relating to field study approvals (i.e., approving body and any reference numbers): This study was authorized by the Ministerio del poder popular de Ecosocialismo, Hábitat y Vivienda (Approval number: 0323) and Territorio Insular Miranda (Approval number 006).

### Data Availability

The following information was supplied regarding data availability: Currently, we don’t have permission from the owner of the data to make it public.
